# Transcriptional Landscape of *Staphylococcus aureus Kayvirus* Bacteriophage vB_SauM-515A1

**DOI:** 10.3390/v12111320

**Published:** 2020-11-17

**Authors:** Maria Kornienko, Gleb Fisunov, Dmitry Bespiatykh, Nikita Kuptsov, Roman Gorodnichev, Ksenia Klimina, Eugene Kulikov, Elena Ilina, Andrey Letarov, Egor Shitikov

**Affiliations:** 1 Federal Research and Clinical Center of Physical-Chemical Medicine of Federal Medical Biological Agency, 119435 Moscow, Russia; herr.romanoff@gmail.com (G.F.); d.bespiatykh@gmail.com (D.B.); kuptsovns@gmail.com (N.K.); grad511@yandex.ru (R.G.); ppp843@yandex.ru (K.K.); ilinaen@gmail.com (E.I.); eshitikov@mail.ru (E.S.); 2 Research Center of Biotechnology of the Russian Academy of Sciences, Winogradsky Institute of Microbiology, 117312 Moscow, Russia; eumenius@gmail.com (E.K.); letarov@gmail.com (A.L.)

**Keywords:** transcriptome analysis, *Kayvirus*, bacteriophage, promoter, terminator, RNAseq, *Staphylococcus aureus*, transcription units

## Abstract

The Twort-like myoviruses (*Kayvirus* genus) of *S. aureus* are promising agents for bacteriophage therapy due to a broad host range and high killing activity against clinical isolates. This work improves the current understanding of the phage infection physiology by transcriptome analysis. The expression profiles of a typical member of the *Kayvirus* genus (vB_SauM-515A1) were obtained at three time-points post-infection using RNA sequencing. A total of 35 transcription units comprising 238 ORFs were established. The sequences for 58 early and 12 late promoters were identified in the phage genome. The early promoters represent the strong sigma-70 promoters consensus sequence and control the host-dependent expression of 26 transcription units (81% of genes). The late promoters exclusively controlled the expression of four transcription units, while the transcription of the other five units was directed by both types of promoters. The characteristic features of late promoters were long -10 box of TGTTATATTA consensus sequence and the absence of -35 boxes. The data obtained are also of general interest, demonstrating a strategy of the phage genome expression with a broad overlap of the early and late transcription phases without any middle transcription, which is unusual for the large phage genomes (>100 kbp).

## 1. Introduction

The viruses of bacteria—bacteriophages—served as key model objects that have helped to gain insight into the nature of genes and the mechanisms of gene expression control, establishing the modern concept of the virus as a transmissive genetic program [1]. Almost immediately after their discovery in the early 20th century, bacteriophages began to be used for the treatment of infectious diseases. However, the introduction of antibiotics overshadowed their therapeutic potential throughout the majority of the world [2,3].

A renewed interest in bacteriophages has been driven by a global antibiotic resistance crisis [4] and by the discovered ecological role of bacteriophage infections in natural microbial populations [5]. Being deeply involved in almost all the aspects of the life and activity of bacteria, bacteriophages cannot be considered merely as “the natural enemies” of their hosts. The interactions between phage and bacterial population in the natural habitats are very complex, normally leading to stable co-existence of the phages and sensitive bacteria [5]. Moreover, many examples of mutually beneficial phage–host interactions have been described [6,7]. These traits of bacteriophage biology make it difficult to use bacteriophages efficiently to combat bacterial infections. To make the phage therapy successful, one needs to artificially set up critical parameters of phage–host interaction to overcome the natural tendency to co-existence and encourage the pathogen eradication. On the other hand, however, the adaptive potential of the phages is equaled to the adaptive power of bacteria. The phages active against almost any resistant strain can be isolated or selected relatively easily. Therefore, phage therapy is considered as a promising approach that may compensate for the loss of antibacterial chemotherapy capacity in the short or medium perspective, unless simpler to use therapeutics [8] will appear on the market.

To achieve this goal, a detailed understanding of the phage infection physiology is crucial. It may not only help to optimize the selection and administration of the naturally occurring bacteriophages for phage therapy but also provides an avenue to the artificial manipulations of the phage genome to obtain improved therapeutic efficacy of the engineered viruses [9].

In the case of *Staphylococcus* infections, the application of bacteriophages as therapeutic alternatives or complements to antibiotic therapy has been evaluated extensively. Staphylophages effectively kill a broad range of methicillin-resistant and susceptible *Staphylococcus* strains and are even effective in eliminating cells in the biofilms [10]. The safety of virulent staphylococcus bacteriophages has been shown in numerous studies on animal models [11,12]. The compassionate use of bacteriophages to treat patients with Methicillin-resistant *Staphylococcus aureus* infections has also been quite successful [13,14]. Moreover, some phage and phage-related products have been approved for human use in several countries [15].

The most promising group of staphylophages belongs to the *Kayvirus* genus of the Twortvirinae subfamily, the Herelleviridae family [16]. Bacteriophages of this group are closely related, are obligatorily lytic, and have a wide host-range. Morphologically, they are characterized by an isometric head and a long contractile tail (170–220 nm).

The overall genome organization of the phages is similar to the other Twortvirinae subfamily members. The average genome size is about 130 kbp with several thousand base pair long direct repeats at the ends. The genome encodes about 200 protein-coding sequences and a few tRNA genes. Genes are tightly packed in modules, which are not clearly separated. Most of the structural and virion-associated proteins were identified by proteomic approaches [17,18]. Recently, the host recognition apparatus, consisting of two receptor-binding proteins, was also described [19,20].

Despite such a large number of studies, the transcriptional organization of bacteriophages of this family is not clear enough. Based on the homology studies, it was suggested that proteins of *S. aureus* kayviruses are translated from over 50 transcripts [21]. Although several putative host-specific promoters and terminators sequences have been identified by in silico analysis of phage K, ISP, vB_SauM-fRuSau02, and A5W genomic sequences [21,22,23], their number and position differ significantly in the studied bacteriophages, despite the overall high similarity of genome sequence and organization. One of the limitations of the existing studies is that software aimed at searching for the bacterial promoters are often used to search for viral promoters. The bacteriophages of the *Kayviruses* genus encode for the phage sigma factor, involved in late transcription. Only recently, a consensus sequence of the late gene promoters has been predicted, which still needs confirmation [22].

Nowadays, the RNA sequencing (RNAseq) approach has been widely applied to study gene expression. In the field of bacteriophage research, this technique has proven to be a good solution for studying phage–host interactions. Recently, the detailed analyses of the bacterial response to phage infection have been described for *Acinetobacter baumannii*, *Campylobacter jejuni* and *Bacillus subtilis* [23,24]. However, only for Giant *Pseudomonas aeruginosa* bacteriophage ϕKZ, the operonic structure has been determined [25].

In the present study, RNA-seq was performed to analyze the transcriptome of virulent staphylophage vB_SauM-515A1 at three time-points representing different infection stages. For the first time, the unique transcriptional landscape of vB_SauM-515A1 was portrayed. Thus, on the basis of experimental data, promoters regulating the expression of early and late genes were identified, and their consensus sequences were established. All the identified transcription units were assigned to early, or late temporal expression classes were determined. This work aimed to expand the understanding of the physiology of the Twortvirinae subfamily staphylophages during phage infection. The mechanisms of regulation of the phage infection can become the fundamental basis for the development of rational phage therapy in diseases involving *Staphylococcus aureus*.

## 2. Materials and Methods

### 2.1. Sample Collection and Growth Conditions

The lytic bacteriophage vB_SauM-515A1 was isolated from the commercial *Staphylococcus* bacteriophage cocktail (batch P332) produced by the Microgen company (Moscow, Russia) using the *Staphylococcus aureus* strain SA515 (spa-type t008) as a host. General characterization of this phage and the conditions used for its cultivation were reported in our previous study [26].

### 2.2. The One-Step Growth Curve of vB_SauM-515A1 Phage

The one-step growth curve of vB_SauM-515A1 was measured as described previously, but the chloroform treatment was added to release the phage progeny from the infected cells prior to their natural lysis [26]. Briefly, cells of *S. aureus* SA515 at the early exponential phase (OD_600_ = 0.12) were infected with vB_SauM-515A1 at an MOI of 0.01 and incubated at 37 °C with shaking (220 rpm). To remove non-adsorbed phages, after 7 min of incubation, the mixture was centrifuged at 10,000× *g* for 3 min, and the pellet was resuspended in 10 mL of the LB broth. The aliquots of 10 μL were taken periodically at 0, 5, 15, 20, 25, 30, 40, 50, 60 and 70 min post-infection and 2% *v/v* of chloroform were added. The samples were briefly vortexed, set aside for 10 min at 37 °C, and centrifuged (10,000× *g* for 5 min). The p.f.u. counts were determined using the double-layer agar plating method. Experiments were carried out in triplicate.

For the transcriptomic analysis, a similar method was employed with slight modifications. The vB_SauM-515A1 phage stock was added to the early exponential phase culture of SA515 bacterial strain (OD600 = 0.1) in order to achieve a MOI value of 10 in 100 mL of LB. Uninfected bacterial cells (OD600 = 0.1) were used as control. The 1 mL samples were collected at 5 min (eclipse phase), 15 min (intracellular accumulation phase) and 30 min (lysis period) post-infection. The whole experiment was triplicated.

### 2.3. Total RNA Extraction

Bacteria were resuspended in 1 mL Trizol (Invitrogen, Carlsbad, CA, USA) and added to a 2 mL Lysing Matrix B (MP Bio, Santa Ana, CA, USA). Cells were disrupted by bead-beating twice for 1 min with a 2 min interval on ice. The suspension was then transferred to a new tube, where chloroform extraction was performed. RNA was precipitated by adding 0.7 volume of isopropanol and washed with 70% ethanol, air-dried, and resuspended in 100 µL Nuclease-free water. DNase treatment was carried out with TURBO DNA-free kit (Thermo Fisher Scientific, Waltham, MA, USA) in volumes of 100 µL and further with the RNase-Free DNase Set (Qiagen, Hilden, Germany) according to the manufacturer’s protocol. RNA cleanup was performed with the RNeasy Mini Kit (Qiagen, Hilden, Germany) according to the RNA Cleanup protocol and stored at −70 °C until further use. The concentration and quality of the total extracted RNA were checked by using the Quant-it RiboGreen RNA assay (Thermo Fisher Scientific, Waltham, MA, USA) and the RNA 6000 pico chip (Agilent Technologies, Santa Clara, CA, USA), respectively.

### 2.4. RNA Sequencing and Analysis

RNA sequencing and analysis were performed as previously described [27] with some modification. Total RNA (1 μg) was used for library preparation. Ribosomal RNA was removed from the total RNA using the RiboMinus Transcriptome Isolation Kit, bacteria (Thermo Fisher Scientific, Waltham, MA, USA) and libraries were prepared using the NEBNext Ultra II Directional RNA Library Prep Kit (NEB, Ipswich, MA, USA), according to the manufacturer’s protocol. Libraries were subsequently quantified by Quant-iT DNA Assay Kit, High Sensitivity (Thermo Fisher Scientific, Waltham, MA, USA). Finally, equimolar quantities of all libraries (12 pM) were sequenced by a high throughput run on the Illumina HiSeq using 2 × 100 bp paired-end reads and a 5% Phix spike-in control. The dataset of RNA-Seq analysis was deposited to the NCBI with the project name PRJNA659920.

Quality control on raw reads was carried out with FASTQC v0.11.5 [28]. Adapters and low-quality sequences were removed with the Trimmomatic v0.33 tool [29]. Reads were aligned against *Staphylococcus* phage vB_SauM-515A1 (GenBank accession number: MN047438.1) and *S. aureus* subsp. *aureus* NCTC 8325 (GenBank accession number: NC_007795.1) using subread-align from the Subread (v.2.0.1) package [30]. featureCounts was used for reads counting [31]. Differential gene expression analysis was performed with the edgeR v.3.30.3 package [32]. For better intrasample comparisons, the trimmed means of M-values (TMM) obtained from edgeR were gene-length corrected (GeTMM) [33]. Genes were considered significantly differentially expressed if they had false discovery rate cutoff (FDR) ≤ 0.01. Bedtools coverage v2.29.2 was used to compute the coverage of sequence alignments. Plots were generated using Gviz v.1.32.0 and ggplot2 v.3.3.2 packages in R v.4.0.2 and further modified with Adobe Illustrator [34,35,36].

For the identification of promoters and terminators, the coverage was visualized and analyzed using the BAC-BROWSER tool [37]. The reads, oriented against the transcription direction (R2 reads), were used to search for the putative transcription start sites (TSSs) prediction, and the transcription-wise R1 reads were used for the transcriptional termination sites (TTSs) prediction. For the identification of promoters, 100 bp regions upstream to up-coverage steps were extracted and analyzed using MEME service. The PWM search (built into the BAC-BROWSER tool) was used to predict promoters across the phage genome. In addition, in silico prediction of the phage promoters was carried out using PhagePromoter v.1.0.1 [38].

For the identification of Rho-independent transcriptional terminators, 100 bp regions around down-coverage steps were extracted, and the secondary structure was calculated using RNA-fold service.

## 3. Results

### 3.1. One-Step Growth Curve of vB_SauM-515A1

**Figure 1:**
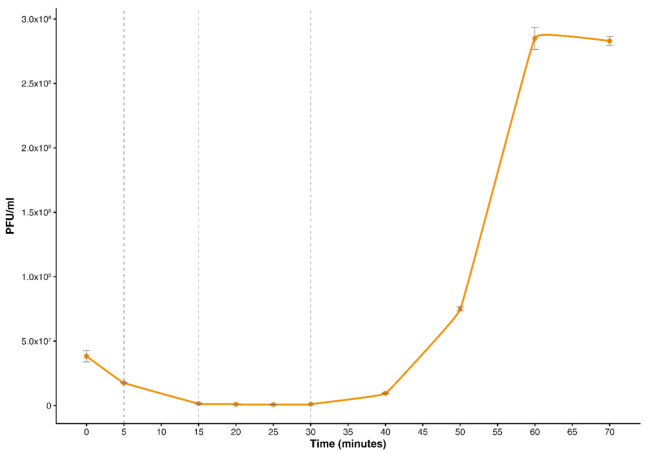
The one-step growth curve of vB_SauM-515A1 bacteriophage. Dotted lines indicate the three sampling time points selected for the transcriptomic analysis.

To better define the timing of the main phases of the lytic infection of *S. aureus* SA515 by the phage vB_SauM-515A1, the single-burst experiment was carried out (Figure 1). The entire replicative cycle of vB_SauM-515A1 in host cells takes about 60 min. According to our data, the latent period ends between 30 and 40 min post-inoculation, after which the bacteriophage particle release started. The number of the phage particles reached the plateau at 60 min post-infection (p.i.), with the burst size of 180 phage particles per infected cell.

### 3.2. Global Transcriptional Changes during Bacteriophage Infection

Based on the one-step growth curve of vB_SauM-515A1, three sampling time points (5 min, 15 min and 30 min) were selected for the transcriptomic analysis (Figure 1). The infected cells were collected at the indicated time moments; their total mRNA was extracted and sequenced. The RNA-seq reads were aligned to the reference bacteriophage genome (MN047438.1) and to the *S. aureus* genome (NC_007795.1). In total, 17,250,861 paired-end reads were mapped to the phage genome and 18,798,359 to the host. At the beginning of the infection, the fraction of reads mapping to the vB_SauM-515A1 phage was 10%; at 15 and 30 min p.i., they accounted for 32% and 56%, respectively, which indicates a progressive takeover of the host transcription machinery by the phage. Accordingly, the amount of host reads decreased from 74% at 5 min p.i. to 27% and 20% at 15 and 30 min, respectively.

All of the vB_SauM-515A1′s ORFs had significant CPM values across all samples determined by filterByExpr function in edgeR (Table S1). The analysis of the intergenic regions showed that the large proportion of the counts (10.6% of all TMM-CPM) was attributed to two long non-coding RNA (lncRNA) regions: lncRNA1 region (570 bp length; pos. 13,730–14,300 in the genome; 2.2% of TMM-CPM) and lncRNA2 region (525 bp length; pos. 38,000–38,525; 8.4% of TMM-CPM). A significant change in lncRNA transcripts abundance was detected during the phage infection: in lncRNA2 FC = 4.68 at 15 vs. 5 min and FC = 45 at 30 vs. 5 min marks; in lncRNA1 FC = 1.7 at 15 vs. 5 min and FC = 5.8 at 30 vs. 5 min marks.

### 3.3. Analysis of Bacteriophage Promoters and Terminators

**Figure 2:**
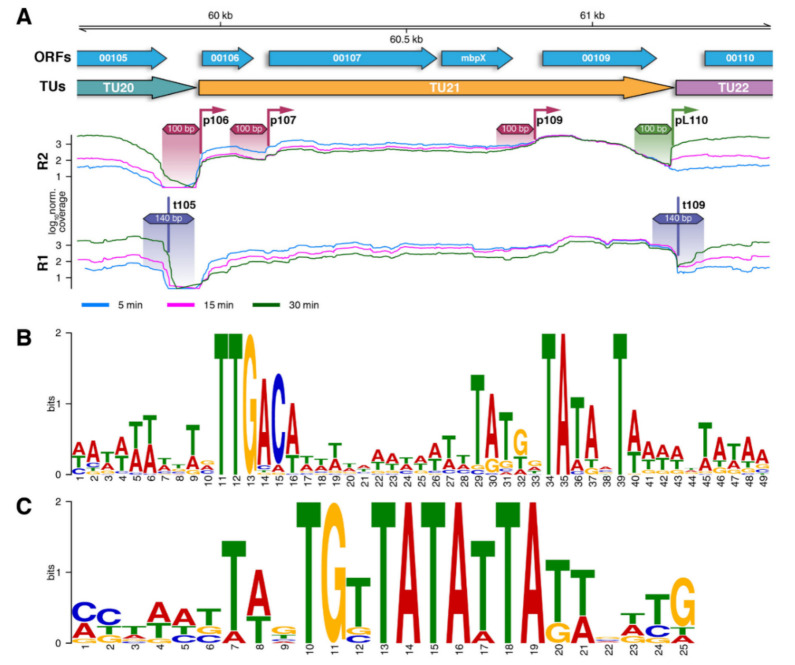
Identification of bacteriophage regulatory elements. (**A**) Search scheme for promoters and transcription terminators. Reverse (R2) and direct (R1) reads were used to predict for TSSs and TTSs, respectively. The color of genes and transcription units corresponds to that in Figure 3; (**B**) consensus sequence logo for early promoters; (**C**) consensus sequence logo for late promoters.

To decipher the mechanism of the phage genes expression regulation more precisely, we searched for the active promoters and transcriptional terminators using RNA-seq data. Putative transcription start sites (TSSs) and transcriptional termination sites (TTSs) were identified by the sharp up- and down-steps of the coverage [39] (Figure 2).

To identify the promoter consensus, the sequence fragments of 100 bp around the TSSs were extracted, and the putative motifs were discovered using MEME [40]. The data for 5 and 15 min post-infection were simultaneously used because we did not find any significant differences specific between these points. In total, 36 manually curated early promoters sequences were selected for the analysis (Table S2). The obtained position weight matrix (PWM) of the early promoter was used to predict promoters across the phage genome. The threshold for the PWM was set to retrieve all sequences that were used to build it. The search yielded additional putative promoters. Some of them correlated with the up coverage steps, which did not pass the initial filtering criteria. The remaining portion of putative promoters did not make any significant contribution to the transcription profile.

Altogether, 58 early promoters were determined for the bacteriophage vB_SauM-515A1 genome (Table S2). The analysis of the promoter region revealed a consensus bacterial promoter of the sigma-70-type consisting of the -10 box (TATANT), the extension (Ext) element (TRTGN) upstream to the -10 box and the -35 box (TTGACW) (Figure 2). The spacer between -10 and -35 was 17 bp-long, a common length for host sigma-70 promoters [41]. Two promoters with 16 and 18 bp spacers were also identified. In addition, the analysis of the promoter region revealed a putative initiating nucleotide at 6 bp position downstream of the -10 box. It allows us to predict spacer length between the -10 box and the TSS to be 5 bp. The preferable nucleotide at this position is T.

Further, we investigated the difference between the groups of promoters, which did or did not influence the transcriptional landscape. Only the minor differences between them were found in the occurrence of particular nucleotides in the up-element (Figure S2)

Late phage promoters were determined by the same method using the data for 30 min p.i. (Figure 2). Only seven regions showed significant up-coverage in steps in the late infection phase. Additional five promoters were found by a search of the PWM across the genome. A conserved TGTTATATTA -10 motif was identified in the late promoters (Figure 2, Table S2). Additionally, active late promoters feature a sort of extension element of TWN consensus upstream of the -10 and TT extension downstream of the -10 motif. The putative initiator nucleotide position is located at 6 bp position after the -10 box precisely as for early promoters and is enriched by G.

A similar approach was conducted to find the transcription terminators. A total of 42 terminators were identified in the phage’s genome (Table S3). Each terminator comprised a GC-rich stem-loop structure followed by the characteristic polyT region, which is common for the Rho-independent terminators [42].

### 3.4. Transcriptional Landscape of vB_SauM-515A1 Phage

**Figure 3:**
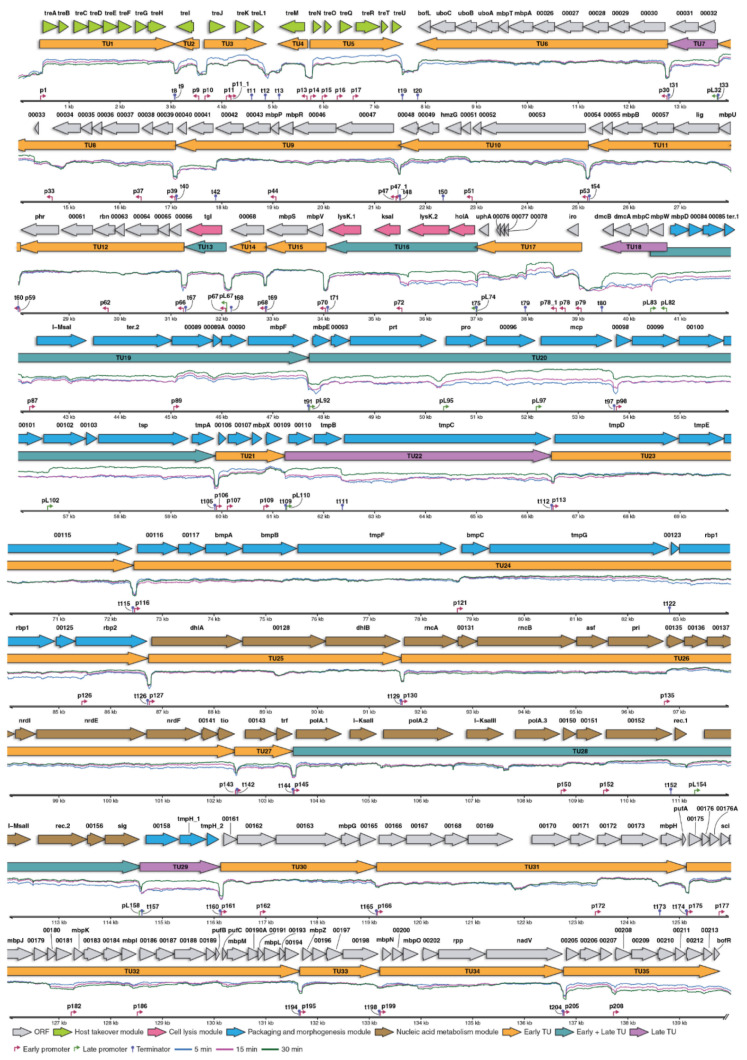
Genetic organization and transcription landscape of the bacteriophage vB_SauM-515A1. The top row shows the ORFs presented by the arrows color-coded according to their functional classification. The second row comprises the transcriptional units (TU) color-coded by their transcriptional response stage. The histogram shows log-transformed transcriptomic read mapping patterns during early (blue), middle (pink) and late (green) infection stages. Axis contains genomic coordinates, early (purple) and late (green) promoters and terminators (blue).

Based on RNA-seq data, promoter and terminator analysis, the transcriptional organization of the vB_SauM-515A1 genome was determined (Figure 3). In total, 35 transcription units (TUs) were identified, of which 20 (57.1%) TUs were transcribed in a positive direction when 15 (42.9%) were transcribed in the negative direction with respect to the standard genomic sequence orientation. Early and late promoters regulated expression of the 26 and 4 TUs, respectively, whereas 5 TUs were under the control of both types of promoters.

It should be noted that at the late stages of infection, we can observe the expression not only from the late promoters but also from early ones due to an ongoing transcription process. At the same time, the design of the experiment does not allow us to say if the expression was indeed going on at a later time point, or we just find the transcripts remaining from the previous time period.

Additionally, we noticed that the difference in the RNA-seq coverage levels between 30 min and 5 min points were always increased for the genes at the end of the operon. This can be explained by the gradual degradation of the transcripts from 5′ ends by some RNA exonuclease activity present in the infected cells.

The largest number of transcription units (N = 26) was controlled by the early sigma-70-dependent promoters and were already expressed at 5 min p.i. They comprised of 193 genes belonging to the following functional categories: host takeover, packaging and morphogenesis, nucleic acid metabolism. Of the identified TUs, five (TU3, TU5, TU15, TU21 and TU32) were abundantly transcribed and had more than one promoter (except TU15) (Figure 3 and Figure S1). TU3 and TU5 are a part of the host takeover module (TU1–TU5) and essential for the initiation of phage infection [43]. Each of the remaining TUs of this module was controlled by a single promoter and did not show a high level of expression. Interestingly, the transcription of TU1 was regulated by the unique promoter with 18 bp spacer. Another highly transcribed unit, TU32, included the largest number of genes (N = 20) and had four promoters (Figure 3). One of the internal promoters of this TU was in front of the *sci* gene, encoding a small protein that specifically inhibits *S. aureus* replicase via binding to a DNA sliding clamp [43].

The late phage promoters directed the TU7, TU18, TU22 and TU29 transcription. We did not find any early promoters for these units, and accordingly, their expression was minimal at 5 and 15 min p.i. (Figure 3, Table S1). Genes of these transcription units were mainly related to the morphogenesis module.

Both early and late promoters are involved in the transcription of the remaining five units (TU13, TU16, TU19, TU20 and TU28) (Figure 3). TU13 is a single-gene TU, controlled by the early promoter p67 and the late promoter pL67. This TU contains the gene *tgI* encoding a lytic enzyme. We observed a moderate expression of this TU throughout the infection, which indicated that both its early and late promoters are weak (Tables S1 and S2). The sequence analysis of the p67 early promoter revealed that in both the -35 (TTGCGT) and -10 (TATGAT) boxes, it differs from the early promoter’s consensus. The late pL67 promoter also contains mismatches if compared to the late promoter’s consensus -10 motif (TGCTATATTA instead of TGTTATATTA).

Other genes of the cell lysis module, *lysK* (major endolysin) and *holA* (holin), were a part of the TU16, also regulated by early and late promoters. Transcription of this TU was completely dependent on the late promoter pL74. However, gene *lysK* is split by the insertion of a mobile type I intron, homologous to the introns annotated in a similar location in some related phages [43]. This intron contains the homing endonuclease gene (*ksaI*) with its own early promoter p72. The transcription from p72 continues till the end of TU16; thus, the 3′- moiety of *lysK* (*lysK.1*) is also transcribed starting from the early stage of infection (Figure 3; Table S1). Such an arrangement of promoters perfectly explains the results of the analysis of differentially expressed genes. The apparent absence of the upregulation of the *ksaI* and *lysK.1* at the late phase compared to *holA* and *lysK.2* is explained by the early transcription of the former genes making these ORFs transcript abundance similar at 5 min and at 30 min.

A similar regulation was observed for TU19, containing morphogenesis and packaging genes (Figure 3). In our interpretation, the transcription of the TU19 genes starts only from the late promoter pL83, whereas the bioinformatically predicted promoters [43] located in the same region as pL83 have only small or no impact on the expression. The -35 box of the pL83 promoter has significant differences from the consensus sequence and therefore was not found by a search for the PWM even with the lowered threshold settings. In turn, the early promoters confirmed in this study are located prior to the genes, encoding endonuclease I-MsaI (prom. p87) and structural protein (prom. p89), respectively (Figure 3). These promoters also did not increase gene expression considerably due to structural changes: p87 had a 16 bp-long spacer, and p89 had differences in the -10 box sequence. (Table S2).

Genes of the morphogenesis and packaging proteins are also found in the TU20. Based on RNA-seq data, most of the TU20 genes were regulated by late promoters and expressed in the late stages of infections (Table S1). A weak early phage promoter (p98; differences in the -35 box sequence) (Table S2) was found in front of ORF00098, encoding a structural protein. This promoter initiates transcription at the early stages of infection and is located immediately after the t97 terminator. In turn, transcription from the late promoters in the late phase of infection almost does not stop at this terminator. It is interesting to note that the expression of the *pro* gene was regulated only by late promoters, which is not consistent with the assumption made earlier [43]. We did not find previously predicted early promoters prior to this gene neither by PWM search nor by RNA-seq data analysis.

TU28 was the last TU that was under the control of both early and late promoters. It contains the genes of nucleic acid metabolism (Figure 3). We identified three early promoters (p145, p150, and p152) for this unit and one late promoter (pL154), located in front of the ORF00154, encoding I-MsaII endonuclease. Although the pL154 had a typical sequence of the phage late promoters, no substantial increase in downstream gene expression was observed at the late stages of infection (Figure 3 and Table S1). We assume this may be related to the genetic background: the sequence of this promoter is in close proximity to the self-splicing *I-msaII* gene, which may affect the level of expression.

## 4. Discussion

*Staphylococcus aureus* bacteriophage vB_SauM-515A1 is a typical member of the *Kayvirus* genus of the *Herelleviridae* family. The genome of the vB_SauM-515A1 is closely related to the genomes of other well-studied kayviruses (vB_SauM_fRuSau (99.82%), MSA6 (99.78%), B1 (99.91%) and JA1 (99.89%) bacteriophages), encodes 238 ORFs and is characterized by a typical structure of all functional modules [44].

To track the transcriptional changes in the bacteriophage throughout the infection cycle, we examined the expression profiles at three time-points after phage infection (5 min, 15 min and 30 min) using RNA-seq analysis. The point 30 min was selected as the last one because, in this time, there is no significant lysis of the cells that could interfere with the results.

Our results demonstrated the presence of 35 independent TUs, where expression was regulated by two separate consensus sequences corresponding for early and late promoters (Figure 3).

The early phage promoters were represented by the strong consensus sigma-70 promoters, common for bacteria, and in particular for *S. aureus* [41,45] (Figure 2). Not all the promoters identified by the consensus sequences significantly influenced the transcriptional landscape. The key differences between strong and weak promoters are likely to be in the architecture of the up-element. However, up-element has too ambiguous consensus and too large size to precisely identify the nucleotide positions, making the most important contribution to the promoter strength. Another important structure is the -10 box, whose sequence has its own features. The limited total number of the promoters does not allow us to make statistically significant conclusions in this regard, but the TAATAT sequence in the -10 box was found only in front of the genes of the host takeover module, whereas the TATACT representation increased towards the end of the bacteriophage genome (Table S2).

In total, 58 early promoters regulated expression of the 193 genes (81% of the genome genes), combined into 31 TUs (Figure 3), suggesting the massive implementation of phage-encoded products in the first stages of infection. The intersection of the obtained promoter sequences with the previously predicted (in silico) for similar bacteriophages showed a high level of convergence, but in the present study, new promoters were found (Table S2). In addition, some promoters could not be found even using the PhagePromoter v.1.0.1 tool (score > 0.5). In turn, PhagePromoter prediction results included about 700 false positives, among which relatively high scored sequences were present, which we could not confirm experimentally in our analysis (Table S4).

As it was previously suggested [43], we did not find middle genes, which was confirmed by transcriptomic data obtained on the 15th minutes after the beginning of the infection. We did not observe any promoters that became activated by this minute in comparison to the 5th minute. The appearance of differentially expressed genes (mainly at the ends of TUs; Table S1) at this stage can be explained by the preferential degradation of the transcripts from the 5′- end, most probably by 5′-RNA exonuclease activity. It is still probable that some transition from early to middle transcription could occur prior to 5 min. For example, in phage T4, the middle gene activity is onset very rapidly after the early transcription start [46]. The resolution of our experiment was not enough to completely exclude such a possibility. However, despite the fact that we have bioinformatically detected 23 potential early promoters that do not change the transcription landscape, the actual transcription at the time points of 5 min and later covers all the ORFs present in the genome. Moreover, the locations of these non-active early promoters are not specifically associated with the functions that could be expected to be pre-early (if we consider as early the genes transcribed at 5 min). Therefore, even if some transcription switch(es) within the early phase detected by our study occurs, the set of the pre-early and early (or early and middle) genes in the phage are completely overlapping. Giving some washing of the initial point due to non-simultaneous infection of the different cells in the culture, more sophisticated experimental approaches would be required to resolve the transcription alteration events within the first 5 min interval.

By the 30th minute of infection, we detected a switch of the transcription phase and expression of late genes in addition to the early without evident shutdown of the later. This can be explained by the fact that bacteriophage anti-sigma factor phage515_00133 (gpORF67 of phage G1) does not block the transcription at most of the promoters. It has been demonstrated that upon expression in this protein binds to the RNA polymerase holoenzyme through the interaction with the sigma subunit, but this binding affects only some strong UP-element-containing promoters [47,48]. Hence, the function of the anti-sigma factor during the phage infection may not be to shut down the early expression but to release a part of the RNA polymerase from the strongly expressed host operons to redirect it for the late TUs of the phage.

The bacteriophage late promoters are significantly less numerous (n = 12) than the early (Figure 3). The analysis revealed their predominant localization within the 36,000–66,000 genomic region encoding virion structural proteins (Figure 3), consistently with the expectations for this class of promoters. The late promoters are characterized by a long -10 box of TGTTATATTA core sequence and show a complete absence of -35 boxes or up-elements (Figure 2). The activity of the late promoter is boosted by two extensions: TWN upstream and TT downstream of the -10 box. Thus, we conclude that the late promoters rely on an alternative phage-specific RNAP sigma factor.

In addition to the described late promoters, we found two more sites in the genome that correspond to the consensus sequence of the late promoter (TGTTATATTA), as well as three sequences that meet the PWM search criteria for late promoters but are classified by us as early promoters. In the first case, the sequences were localized after the gene *hmzG* and before the gene *bmpC* and did not give significant increases in expression. Additionally, one of the sequences was located just before the transcription terminator t33. In the second case, promoters p33, p116 and p150 met the criteria of both early and late promoters. The distinctive feature of the presented promoters was the presence of the -35 box in their sequences. Such a combined promoter can be continuously active through the phage life cycle, but in our experiment, we found no increase in the expression of the underlying genes. This suggests that the presence of the -35 box will most likely determine the regulatory capacity of the promoter.

The late promoter pL154 located within the TU28 also did not show any marked step-up of the RNA-seq coverage (Figure 3) despite the fact that its sequence perfectly matches the late promoter consensus. At the moment, we do not have any consistent explanation for this controversy. Interestingly, both pL154 and ORF00154 are located within the predicted self-splicing type I intron [43]. The splicing should remove the TSS-adjacent region, at least from the early transcripts containing both ends of the intron. It is not clear if the late transcripts may also be affected due to the trans-splicing. Therefore, the instability of the transcripts fragment around the TSS of the pL154 promoter may interfere with the measurement of the expression of this region based on the RNA-seq coverage. It is also possible that, despite its “good” sequence, the pL154 promoter is weakened by an unidentified factor. The evaluation of the true pL154 activity and the influence of the intron splicing on it requires complex experimentation that falls out of the scope of this study.

In addition to the expression of the coding part of the vB_SauM-515A1 genome, RNA-seq data allowed estimating the level of transcription of non-coding RNA. Thus, two non-coding regions (lncRNA1 region (570 bp) and lncRNA2 region (525 bp)) were detected for the minus DNA chain of vB_SauM-515A1. These regions were in close proximity to tRNA genes. The lncRNA1 region was located immediately after the tRNA-Met gene (TU8), while the lncRNA2 region (TU17) preceded the tRNA genes (tRNA-Trp, tRNA-Phe, tRNA-Asp).

Several non-coding RNA sequences were found precisely in the tRNA region of *Pseudomonas aeruginosa* and *Lactobacillus* bacteriophages [49,50]. Regarding the regulation of the lncRNA1 and lncRNA2 regions transcription, the lncRNA1 region was transcribed directly after the tRNA-Met gene from the main promoter of TU8, and no additional promoter was found to explain such a high level of transcription of this region. The lncRNA2 region transcription was controlled by a typical early promoter (p78); no features in its sequence indicating its increased efficiency were found. The literature describing bacteriophage non-coding RNA is very limited. Attempts at establishing their functions have been made, but there is no clarity on this issue so far. Nevertheless, very high levels of their transcription shown in this study (lncRNA1 region −2% of CPM; lncRNA2 region −9% of CPM) indicate the potential importance of these regions in the life cycle of kayviruses.

## 5. Conclusions

In summary, this study represents the first reported whole-transcriptome sequencing analysis of the staphylococcal *Kayvirus* genus phage during the infection process. We described a transcription landscape of a bacteriophage consisting of 35 transcription units. Most of the units of the bacteriophage were already transcribed on the fifth minute of infection from early promoters, the exact structure of which was also determined during the study. In turn, late promoters either increased the expression of early genes or served as drivers for later genes.

## Supplementary Materials

The following are available online at https://www.mdpi.com/1999-4915/12/11/1320/s1, Figure S1: Bar graph showing the gene length corrected trimmed mean of M-values (GeTMM-CPM) of 35 transcriptional units (TU) from RNA-seq analysis at three time-points during vB_SauM-515A1 phage infection. Data are presented as the mean of three biological repeats ± SD. Figure S2: Consensus sequence logo for early promoters correlated and not correlated with the up coverage steps. Table S1: Dataset of DEG analysis from the study. Table S2: Early and late promoter sequences of the vB_SauM-515A1 bacteriophage. Table S3: Putative terminator sequences identified in the vB_SauM-515A1 genome. Table S4: Comparison of identified promoters-with-promoters predicted by PhagePromoter.
